# Aspirin induces autophagy *via* inhibition of the acetyltransferase EP300

**DOI:** 10.18632/oncotarget.25364

**Published:** 2018-05-15

**Authors:** Francesca Castoldi, Federico Pietrocola, Maria Chiara Maiuri, Guido Kroemer

**Affiliations:** Guido Kroemer: Gustave Roussy Cancer Campus, Villejuif, France; INSERM, U1138, Paris, France; Equipe 11 labellisée par la Ligue Nationale contre le Cancer, Centre de Recherche des Cordeliers; Paris, France; Université Paris Descartes/Paris V, Sorbonne Paris Cité, Paris, France; Université Pierre & Marie Curie, Paris, France; Université Paris-Sud/Paris XI, Faculté de Médecine, Kremlin-Bicêtre, France; Metabolomics and Cell Biology Platforms, Gustave Roussy Cancer Campus, Villejuif, France; Pôle de Biologie, Hôpital Européen Georges Pompidou, AP-HP, Paris, France; Karolinska Institute, Department of Women’s and Children’s Health, Karolinska University Hospital, Stockholm, Sweden

**Keywords:** aging, AMPK, cancer, inflammation, longevity, Autophagy

Aspirin (acetylsalicylate) is the chemically synthesized prodrug of salicylate and is therapeutic used since the end of the 19th century. Until nowadays, aspirin is one the most widely used medications globally. As for many other drugs, the mode of action of aspirin has not been known when it was introduced into human medicine as a nonsteroidal anti-inflammatory drug to treat pain, fever and inflammation. Aspirin is also widely used for the prevention of heart attack, ischemic stroke and blood clots. Epidemiological studies revealed that daily long-term use of aspirin for more than a decade reduces the incidence of most carcinomas, in particular gastrointestinal cancers. [1] Moreover, long-term use of aspirin is linked to a reduction of cardiovascular morbidity and mortality. [2] As such, aspirin may be considered as the one single medication that has the best long-term disease prophylactic effects on humans. Unfortunately, aspirin may cause stomach ulcers and bleeding, a potentially lethal complication that precludes the recommendation of aspirin use by the general population.

The principal mode of action of aspirin was thought to reside in the irreversible inactivation of cyclooxygenase-1 (COX-1; officially known as prostaglandin-endoperoxide synthase, PTGS1) and the inhibition of COX-2/PTGS2, thus suppressing the production of pro-inflammatory and thrombocyte-aggregating prostaglandins and thromboxanes. The discovery of this mode of action (in 1971) led the British pharmacologist John Robert Vane to earn the Nobel Prize in 1982. [3]. Moreover, it triggered the febrile research for specific COX-1 and COX-2 inhibitors, which however have failed to yield similar pro-health effects as aspirin, and several specific COX-2 inhibitors turned out to increase the risk of heart attack and stroke, leading to their withdrawal from the market. [4] Based on the failure of specific COX-1/COX-2 inhibitors to mimic the broad health-improving effects of aspirin, other modes of action have been proposed. These include, uncoupling of oxidative respiration through proton transport on the inner mitochondrial membrane [5], inhibition of the pro-inflammatory transcription factor NF-kB [6] and allosteric activation of AMP-activated kinase (AMPK). [7]

**Figure 1:**
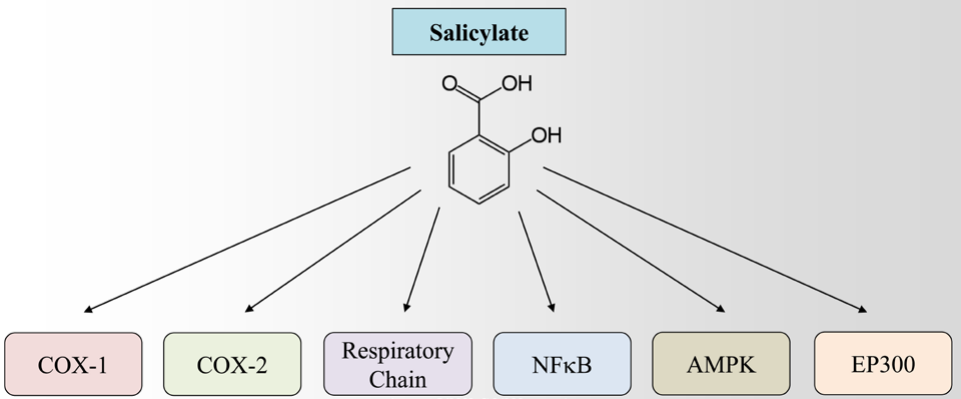
Currently described mechanisms underlying the mode of action of salicylate The aspirin active metabolite salicylate can modulate the activity of several molecular targets including cyclooxygenase 1 (COX-1), cyclooxygenase 2 (COX-2), NFκB (nuclear factor kappa-B), AMP-activated protein kinase (AMPK), mitochondrial respiratory chain and histone acetyltransferase p300 (EP300).

Recently, we have discovered another plausible mechanism that explains the broad pro-health activity of aspirin and its active metabolite salicylate (Fig. 1). Indeed, salicylate competitively inhibits the binding of acetyl coenzyme A (AcCoA) to E1A-associated protein p300 (where E1A = adenovirus early region 1A), best known as EP300, thus inhibiting its acetyltransferase activity. In this respect, aspirin resembles other EP300 inhibitors such as the natural polyamine spermidine, the green tea flavonoid epigallocatechin gallate, and the cashew nut compound anacardic acid, which is a salicylate derivative. By virtue of inhibiting EP300 (and perhaps other acetyl transferases), aspirin inhibits the acetylation of cellular proteins with the final result that autophagy is induced. [8]

The physiological, evolutionarily conserved (from yeast to humans) stimulus of autophagy is nutrient scarcity. Insufficient external supply of carbohydrates, proteins or lipids obliges cells to mobilize their own reserves, a process that is best achieved by the macroautophagic sequestration of cytoplasmic structures, followed by the lysosomal digestion of macromolecules into micromolecules that can be used to fuel the bioenergetic demand of the cell and to synthesize molecules required for stress adaptation. The molecular link between nutrient depletion and autophagy induction apparently involves a depletion of the cytosolic pool of AcCoA, thus ultimately causing cellular protein deacetylation for the simple reason that AcCoA is the sole acetyl donor for acetyltransferases including EP300. This protein deacetylation affects hundreds of different proteins including multiple regulators and effectors of autophagy, thus setting of the autophagic cascade. [9]

Accordingly, mutation of EP300 resulting in reduced salicylate binding causes aspirin to lose its pro-autophagic activity. Deletion or depletion of EP300 by genetic means is epistatic to salicylate with respect to autophagy induction, both *in vitro*, in mammalian cells, and *in vivo*, in the nematode *Caenorhabditis elegans*. This contrasts with the fact that cells lacking AMPK are still able to mount an autophagic response upon exposure to salicylate

All these results are compatible with the interpretation that salicylate induces autophagy via the inhibition of EP300 rather than the activation of AMPK. [8]

Autophagy has prominent antiaging, antineoplastic and anti-arteriosclerotic effects, explaining the broad pro-health effects of EP300 inhibitors such as spermidine. [10]

It is tempting to speculate, yet remains to be demonstrated in further detail, that aspirin may mediate its anticancer and anti-cardiovascular disease effects also via the induction of autophagy.
